# Mapping the Research Trends and Knowledge Structure of Traditional Chinese Medicine for Musculoskeletal Disorders: A Bibliometric Analysis

**DOI:** 10.7759/cureus.112758

**Published:** 2026-07-15

**Authors:** Chunxue Jiang, Yiyuan Cui, Ke Li, Shiren Luo, Pengge Cheng, Ruiqi Li, Huanhuan Ma, Yaohua Liu

**Affiliations:** 1 Laboratory of Clinical Pharmacy, Henan Provincial Orthopedic Hospital, Henan Luoyang Orthopedic Hospital, Zhengzhou, CHN; 2 Department of Pharmacy, Henan Provincial Orthopedic Hospital, Henan Luoyang Orthopedic Hospital, Zhengzhou, CHN; 3 Medicine, Gansu University of Chinese Medicine, Gansu Pharmaceutical Industry Innovation Research Institute, Lanzhou, CHN

**Keywords:** citespace, musculoskeletal disorders, research trends, traditional chinese medicine, vosviewer

## Abstract

Musculoskeletal disorders represent a major global health burden and substantially impair the quality of life. Traditional Chinese medicine (TCM), characterized by its multi-component and multi-target therapeutic properties, has been widely used in the prevention and treatment of major musculoskeletal disorders. However, a comprehensive evaluation of the global research landscape and emerging trends in this field remains lacking. Therefore, we retrieved publications related to TCM therapy for major musculoskeletal disorders from the Web of Science Core Collection (WoSCC) published between January 2016 and August 24, 2025, and performed a bibliometric analysis using CiteSpace and VOSviewer to evaluate publication trends, leading countries and institutions, influential journals and authors, co-cited references, and research hotspots. A total of 781 publications were included. The annual number of publications showed a continuous upward trend over the past decade. China contributed the largest number of publications, whereas the United States demonstrated greater academic influence based on average citations. The Journal of Ethnopharmacology and Frontiers in Pharmacology were identified as the leading journals in this field, while Chunyan Yan and Xin-Sheng Yao were the most productive authors. Co-citation and keyword analyses identified osteoporosis, knee osteoarthritis, active compounds, and molecular signaling pathways, particularly NF-κB, as the major research hotspots. Furthermore, citation burst analysis revealed a shift from studies of single herbal medicines toward TCM formulas, accompanied by the increasing application of network pharmacology and molecular docking, highlighting a transition from efficacy-oriented research to mechanism-based investigations. Overall, this study provides a comprehensive overview of the knowledge structure and research trends of TCM for major musculoskeletal disorders. The findings provide valuable references for future research and suggest that muscle diseases and spinal disorders remain underrepresented and deserve greater attention in future investigations.

## Introduction and background

Musculoskeletal disorders represent a significant global health burden that severely influences the quality of life. The incidence is rising markedly due to population aging and changes in modern lifestyles. According to WHO statistics, approximately 1.71 billion people worldwide are affected by musculoskeletal disorders annually, with nearly half of all adults in the United States experiencing musculoskeletal disorders [1]. Common examples include fractures, cervical spondylosis, lumbar disc herniation, and arthritis. These conditions not only cause physical pain and functional limitations but may also lead to long-term disability, imposing a substantial burden on both families and society; notably, back and neck pain rank fourth in the global disability-adjusted life years (DALYs) ranking [2].

Traditional Chinese medicine (TCM) has been widely used for the prevention and treatment of musculoskeletal disorders for thousands of years and has attracted increasing research interest because of its multi-component and multi-target therapeutic characteristics. Previous studies have demonstrated that 42 active TCM compounds and six representative herbal formulas exhibit therapeutic potential for osteoarthritis through anti-inflammatory effects, cartilage protection, and regulation of signaling pathways, including MAPK and NF-κB [3]. With the growing application of modern experimental techniques and molecular biology, increasing attention has been directed toward elucidating the pharmacological mechanisms and clinical applications of TCM for musculoskeletal disorders. Consequently, research interest in this field has increased substantially over the past decade, highlighting the need for a comprehensive bibliometric evaluation of research trends, knowledge structure, and emerging hotspots in this field.

Bibliometric analysis is a quantitative approach for evaluating scientific literature and has been widely used to characterize the development of research fields through publication trends, influential contributors, collaboration networks, co-cited references, and research hotspots. By systematically analyzing the published literature, bibliometric methods provide valuable insights into the knowledge structure and emerging research directions within a specific field. Although numerous studies have investigated the clinical application and pharmacological mechanisms of TCM for musculoskeletal disorders, a comprehensive understanding of the global research landscape and its evolving trends remains lacking. Therefore, we conducted a bibliometric analysis of publications related to TCM therapy for major musculoskeletal disorders retrieved from the Web of Science Core Collection (WoSCC) between January 2016 and August 24, 2025. This study aimed to evaluate research productivity, identify major contributors, visualize the knowledge structure through co-citation and keyword analyses, and reveal emerging research hotspots and future development trends. Particular attention was paid to identifying emerging methodological trends, especially the increasing application of network pharmacology and molecular docking in TCM research for major musculoskeletal disorders. WoSCC was selected because of its high-quality indexing, standardized bibliographic records, and widespread use in bibliometric studies, making it a reliable database for quantitative literature analysis.

## Review

Methods

Data Acquisition

The Web of Science Core Collection (WoSCC) was selected as the data source because it provides standardized bibliographic records, comprehensive citation information, and has been widely recognized as one of the most reliable databases for bibliometric studies. The literature search was conducted on August 24, 2025, and publications indexed between January 1, 2016, and August 24, 2025, were retrieved. As the literature search was completed before the end of 2025, publications from 2025 represent partial-year data and were interpreted accordingly during data analysis. The search strategy focused on publications related to traditional Chinese medicine (TCM) therapy for major musculoskeletal disorders. The search query was as follows: TS = (("orthopedic diseases" OR "osteoarthritis" OR "osteoporosis" OR "bone fracture" OR "spinal stenosis" OR "intervertebral disc degeneration") AND ("traditional Chinese medicine" OR "TCM" OR "Chinese herbal medicine")). The complete search strategy is provided in the Appendix.

Inclusion and Exclusion Criteria

The literature was screened according to predefined inclusion and exclusion criteria. The inclusion criteria were as follows: (1) full-text publications related to traditional Chinese medicine (TCM) therapy for major musculoskeletal disorders; (2) articles and reviews published in English; and (3) publications indexed between January 1, 2016 and August 24, 2025. The exclusion criteria were as follows: (1) publications unrelated to TCM therapy for major musculoskeletal disorders; (2) conference abstracts, news reports, briefings, book chapters, editorial materials, corrections, and other non-research publication types; and (3) non-English publications. Following the application of these criteria, 781 publications were included in the subsequent bibliometric analysis.

Data Analysis and Visualization

**Figure 1:**
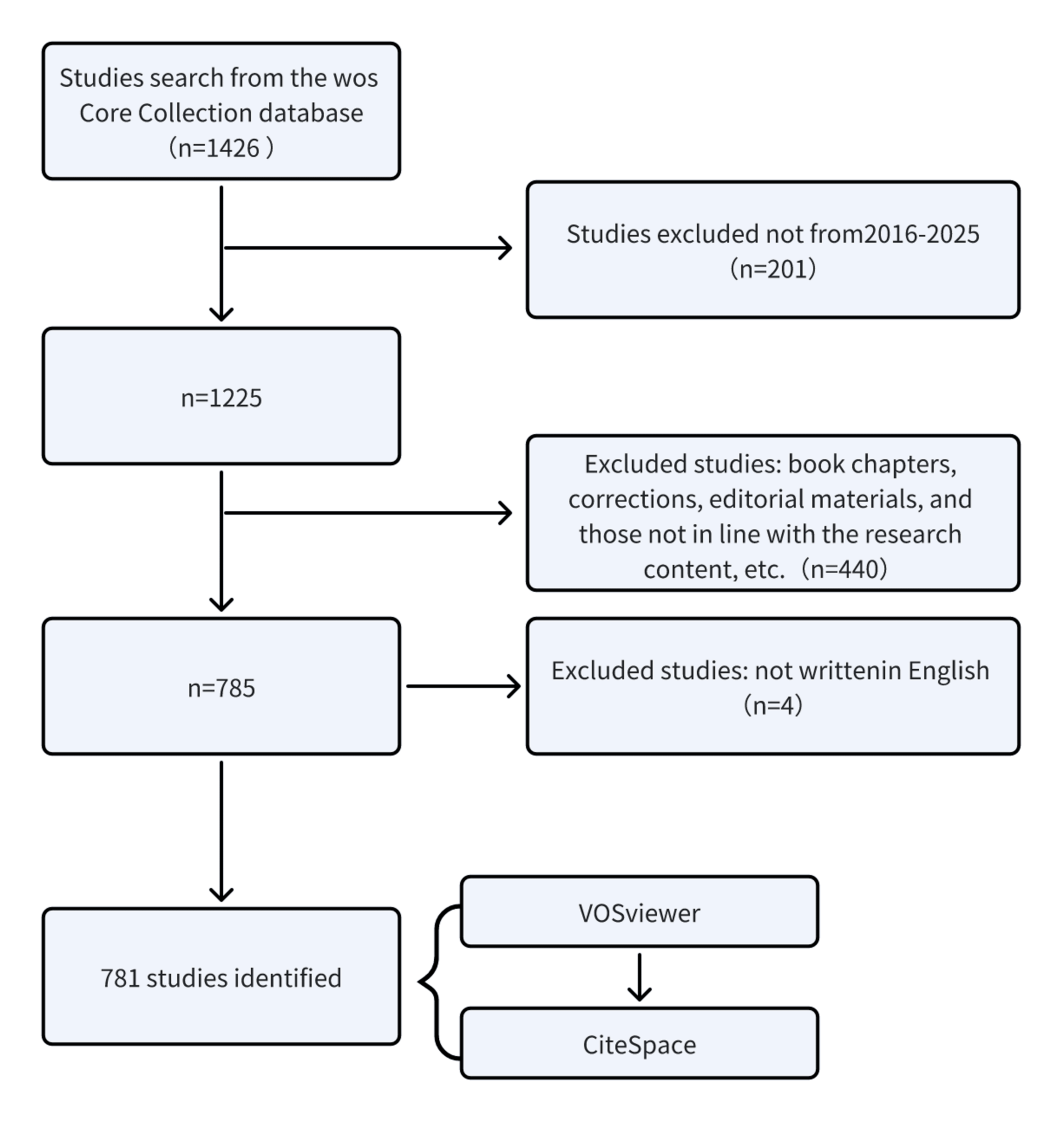
Flowchart of literature search

The annual publication trends and the distribution of publications by country were analyzed and visualized using GraphPad Prism (version 8.0.2). Bibliometric analyses and knowledge visualization were performed using CiteSpace (version 6.1.R6, 64-bit Basic) and VOSviewer (version 1.6.18). In CiteSpace, the time span was set from 2016 to 2025 with one year per slice. The g-index (k = 25) was used as the selection criterion, and the default parameters (LRF = 3.0, L/N = 10, LBY = 5, e = 1.0) were applied. No pruning was performed during network construction. CiteSpace was used for co-citation analysis, clustering analysis, timeline visualization, and keyword burst detection, whereas VOSviewer was used to construct and visualize collaboration networks among countries, institutions, authors, and keywords. These analyses were performed to identify research hotspots, knowledge structures, and emerging trends in the field. The literature identification and study selection process is presented in Figure 1. This study employed descriptive bibliometric methods based on publication metadata and network visualization. Therefore, no inferential statistical analyses (e.g., hypothesis testing, p-values, or confidence intervals) were performed.

Results

**Figure 2:**
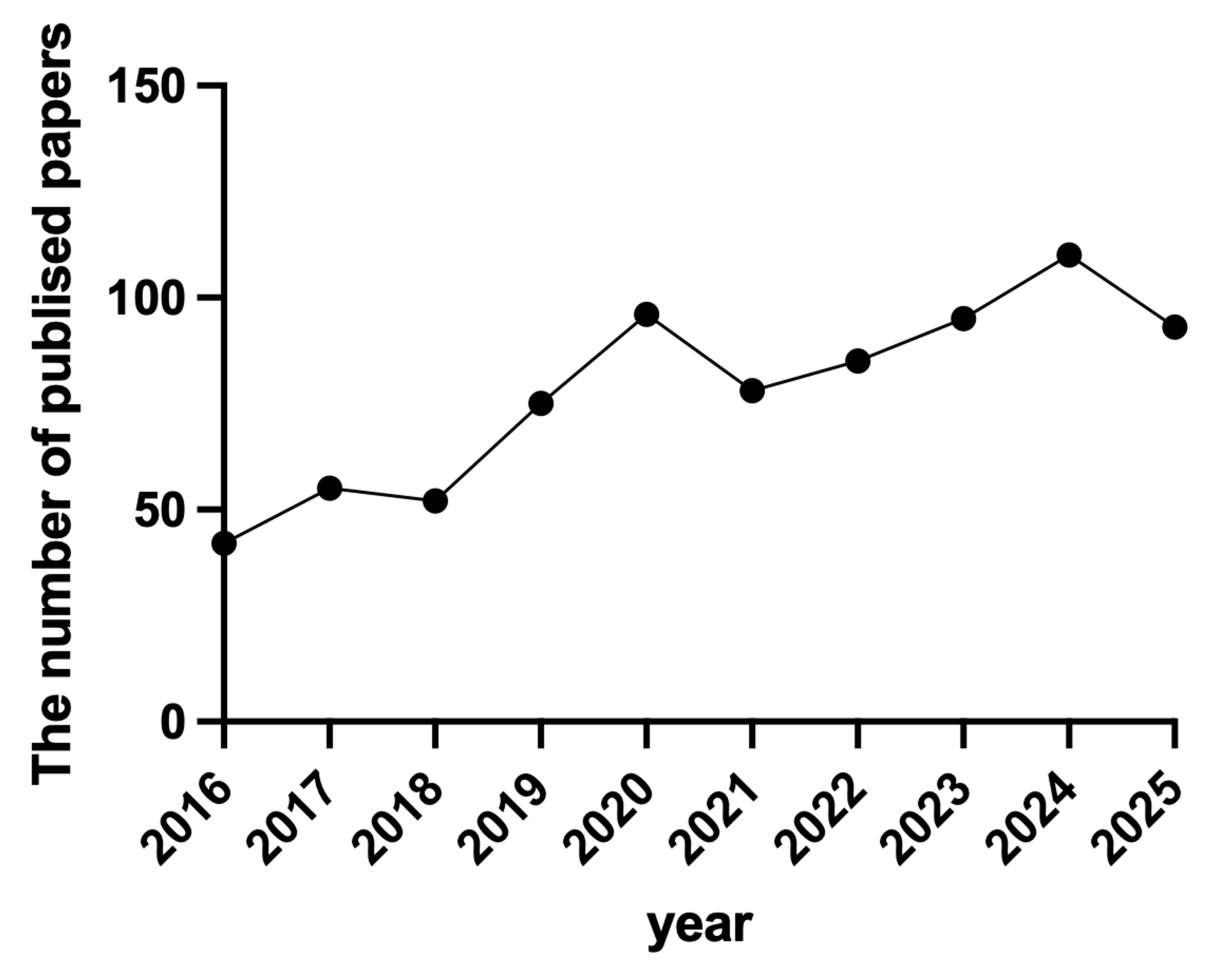
Annual publication volume chart

A total of 781 publications related to TCM therapy for major musculoskeletal disorders were retrieved from the Web of Science Core Collection (WoSCC), involving 4581 authors from 2382 institutions across 58 countries and regions. As shown in Figure 2, the annual number of publications showed an increasing trend between 2016 and 2025, suggesting growing research interest in this field. Since the literature search was conducted on August 24, 2025, the publication count for 2025 represents partial-year data and should be interpreted accordingly.

Countries and Institutions

**Figure 3:**
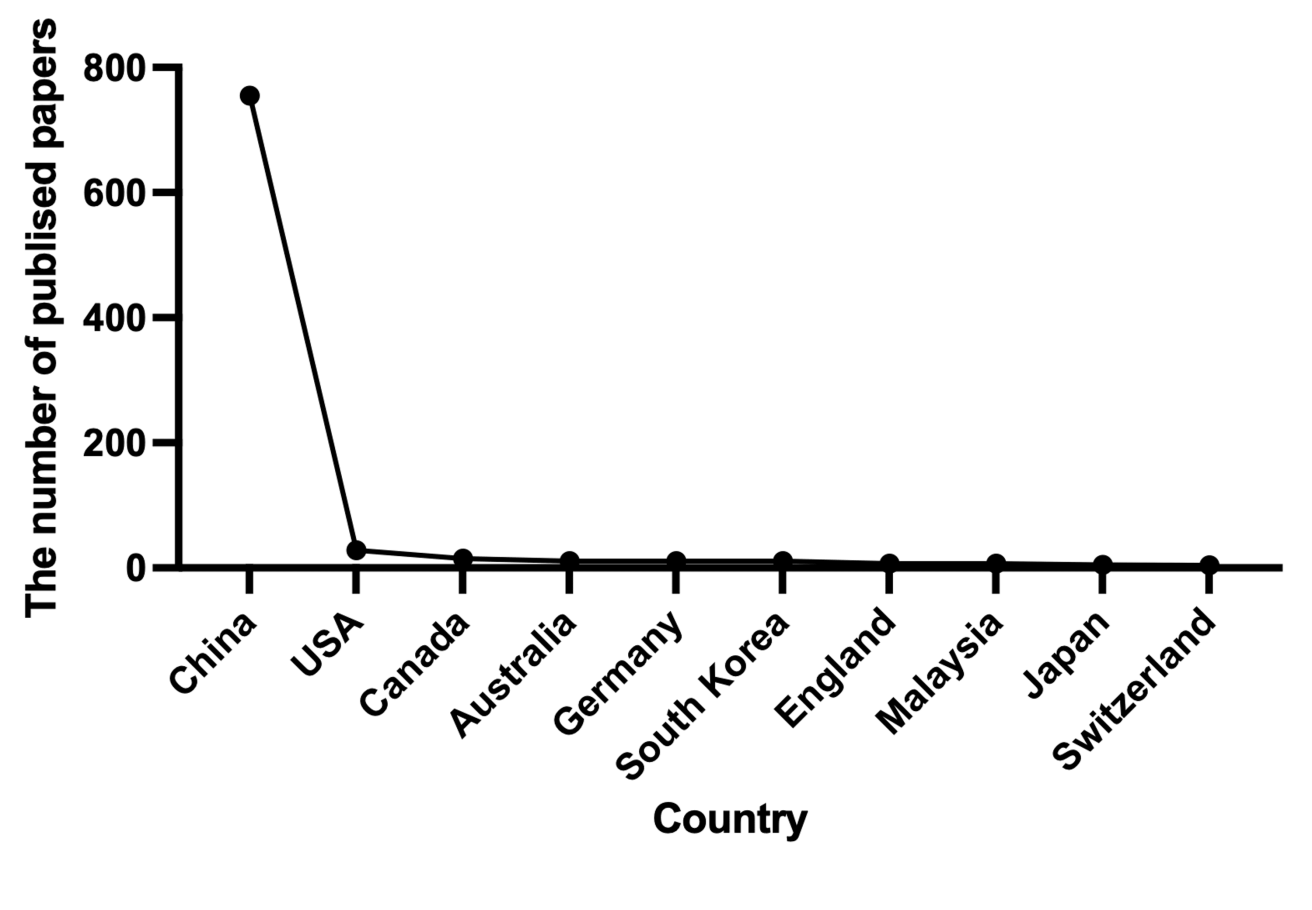
Number of documents issued by country

A total of 58 countries and regions contributed to research on TCM therapy for major musculoskeletal disorders. The 10 most productive countries are presented in Figure 3 and Table 1, with China, the United States, Canada, Australia, and Germany ranking among the top five. China ranked first in publication output (755 publications) and total citations (13,209), whereas the United States had the highest average citation (32.04), indicating greater citation impact. Although China produced the largest number of publications, its average citation (17.50) was lower than that of the United States.

**Table 1:** Document publication volume by country

Rank	Country/region	Article counts	Percentage (%)	Citation	Average citation
1	China	755	96.67%	13209	17.50
2	USA	28	3.59%	897	32.04
3	Canada	15	1.92%	571	38.07
4	Australia	11	1.41%	236	21.45
5	Germany	11	1.41%	349	31.73
6	South Korea	11	1.41%	101	9.18
7	England	7	0.90%	480	68.57
8	Malaysia	7	0.90%	62	8.86
9	Japan	5	0.64%	36	7.20
10	Switzerland	4	0.51%	216	54.00

**Figure 4:**
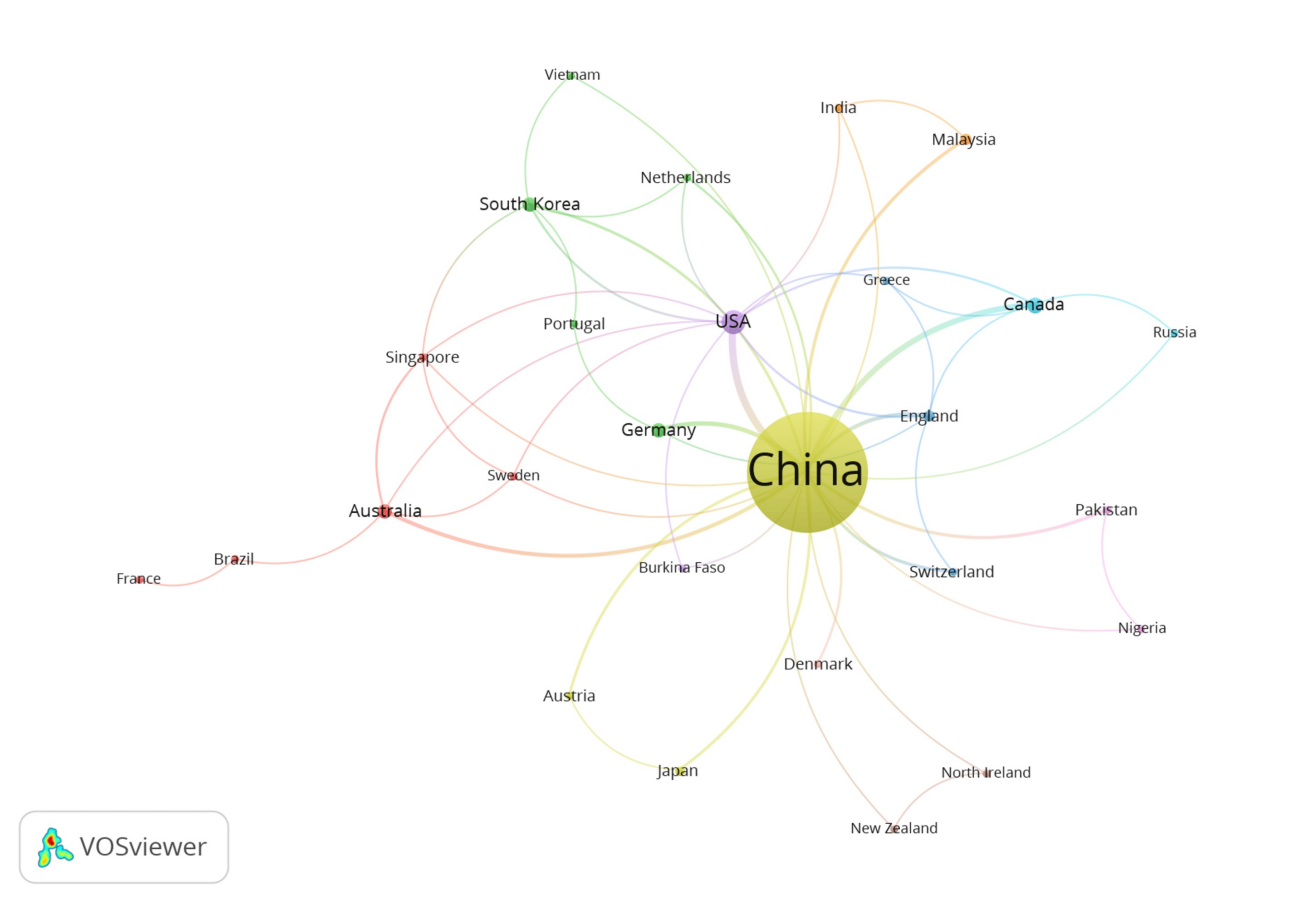
Network diagram of national cooperation Diagram was created by authors using VOSviewer (version 1.6.18).

Figure 4 illustrates the collaboration network among countries. China and the United States, the two most productive countries, showed extensive international collaboration. The United States maintained close collaborations with England and South Korea, whereas China showed strong collaborative relationships with Germany, England, and Canada. Overall, China was the leading contributor in terms of publication output, while the United States demonstrated greater international citation impact.

**Figure 5:**
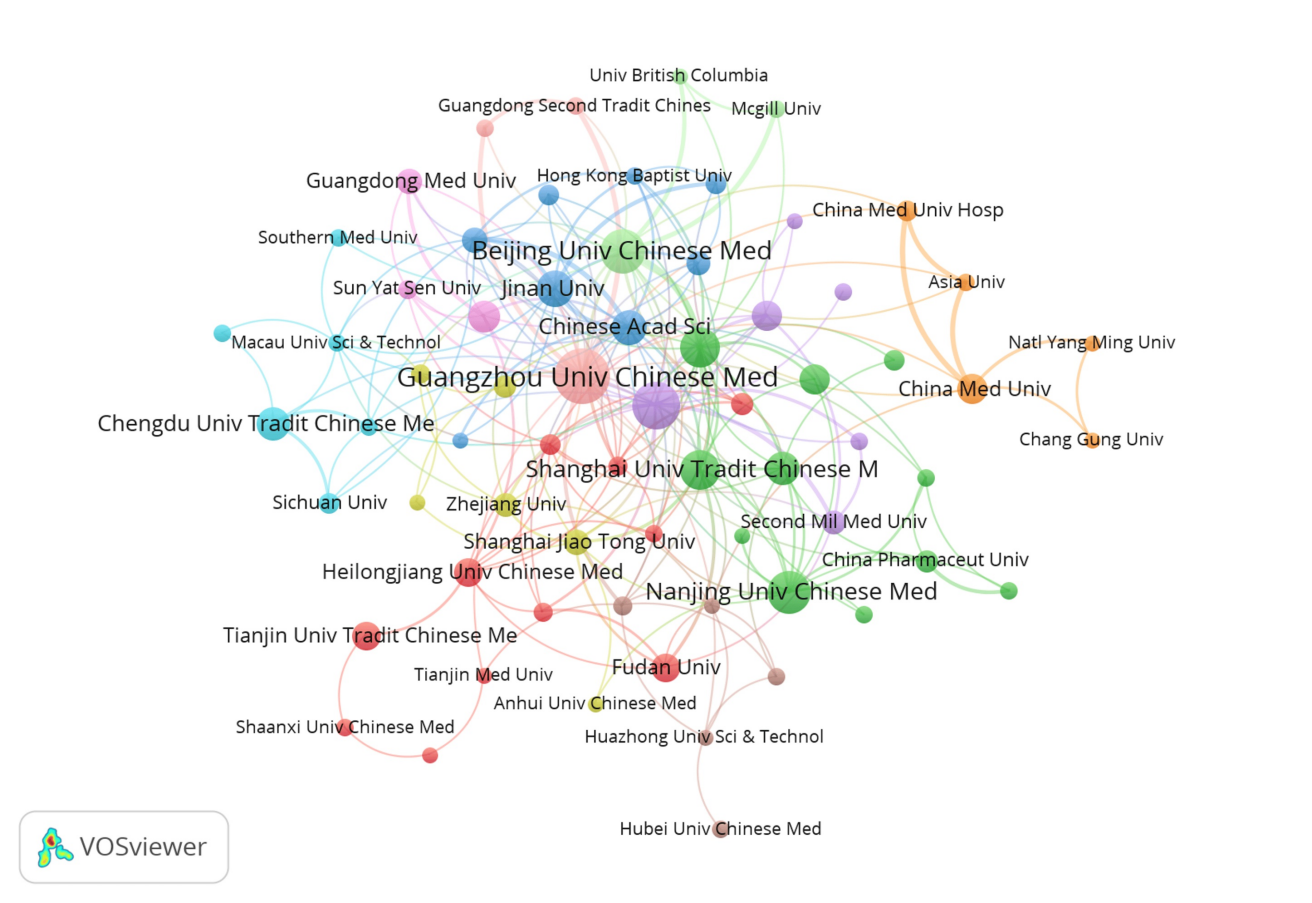
Network diagram of institutional cooperation Diagram was created by authors using VOSviewer (version 1.6.18).

A total of 2382 institutions contributed to publications on TCM therapy for major musculoskeletal disorders. Guangzhou University of Chinese Medicine ranked first with 61 publications, receiving 775 citations with an average citation of 12.70 per publication. Zhejiang Chinese Medical University ranked second with 47 publications and 980 citations, corresponding to an average citation of 20.85 per publication. Beijing University of Chinese Medicine ranked third with 39 publications and 966 citations, with an average citation of 24.77 per publication. As shown in Figure 5, the VOSviewer collaboration network indicated that most institutions tended to collaborate more frequently with institutions within the same country than with those from other countries. The institutional publication and citation data are summarized in Table 2.

**Table 2:** Top institutional publications and citations

Rank	Institution	Country	Number of papers	Total citations	Average citation
1	Guangzhou Univ Chinese Med	China	61	775	12.70
2	Zhejiang Chinese Med Univ	China	47	980	20.85
3	Beijing Univ Chinese Med	China	39	966	24.77
4	Nanjing Univ Chinese Med	China	37	622	16.81
5	China Acad Chinese Med Sci	China	31	603	19.45
6	Shanghai Univ Tradit Chinese Med	China	31	623	20.10
7	Jinan Univ	China	27	426	15.78
8	Chinese Acad Sci	China	26	707	27.19
9	Chengdu Univ Tradit Chinese Med	China	22	473	21.50
10	Shandong Univ Tradit Chinese Med	China	22	406	18.45

Journals

**Figure 6:**
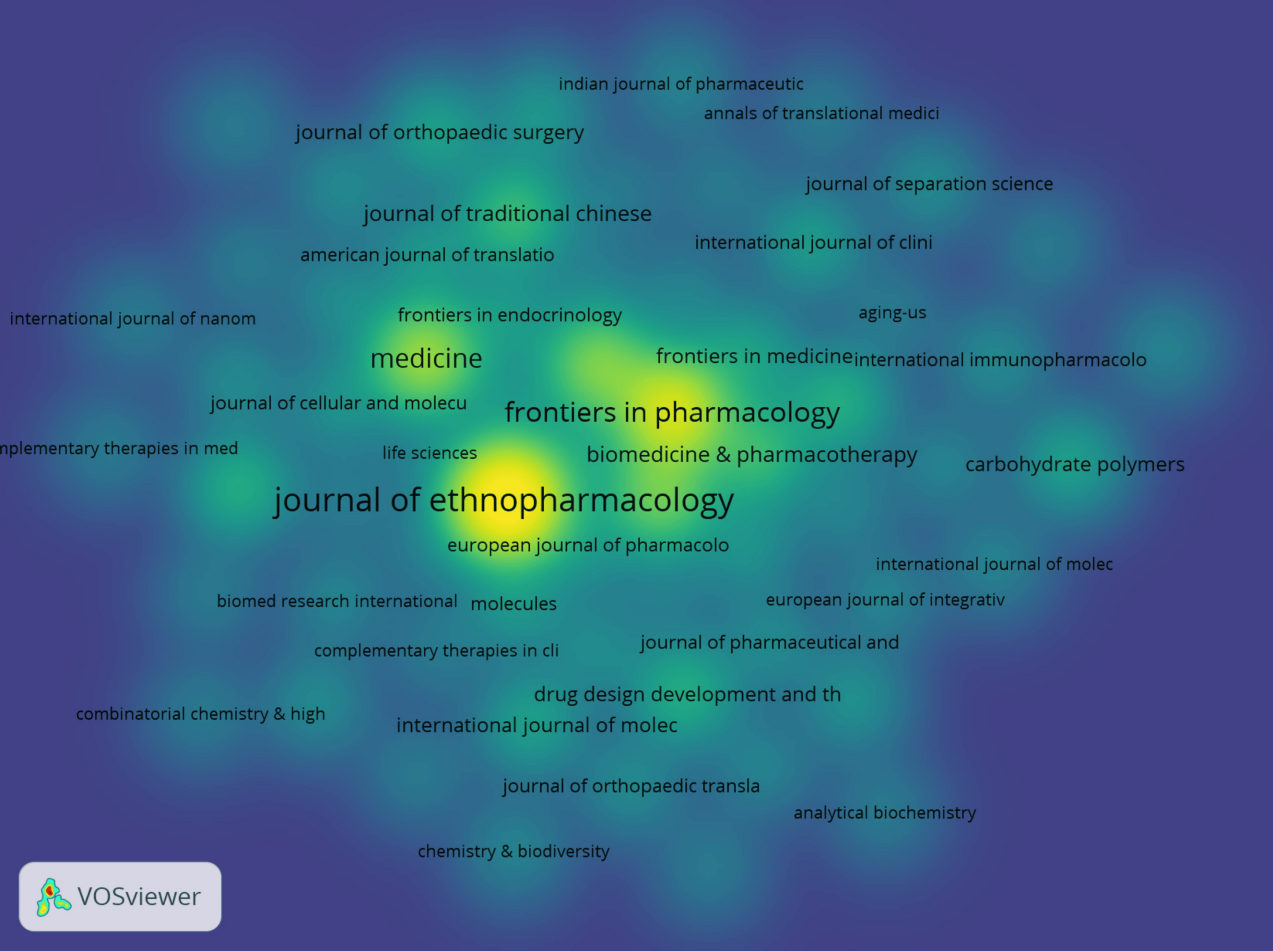
Publication density map of magazines Density map was created by authors using VOSviewer (version 1.6.18).

Figure 6 illustrates the journal density map, and Table 3 summarizes the 10 most productive journals in this field. The Journal of Ethnopharmacology published the largest number of articles (91 publications, 11.65%), followed by Frontiers in Pharmacology (53 publications, 6.79%), Medicine (35 publications, 4.48%), and Evidence-Based Complementary and Alternative Medicine (28 publications, 3.59%). Among the top 10 journals, Phytomedicine had the highest impact factor (8.3), and 80% of these journals were classified as Q1 or Q2 journals.

**Table 3:** Journal publication volume IF - impact factor

Rank	Journal	Article counts	Percentage (781）	IF (2024)	Quartile in category
1	Journal of Ethnopharmacology	91	11.65%	5.4	Q1
2	Frontiers in Pharmacology	53	6.79%	4.8	Q1
3	Medicine	35	4.48%	1.4	Q2
4	Evidence-Based Complementary and Alternative Medicine	28	3.59%	-	Q3
5	Phytomedicine	25	3.20%	8.3	Q1
6	Biomedicine & Pharmacotherapy	16	2.05%	7.5	Q1
7	Journal of Traditional Chinese Medicine	16	2.05%	2.2	Q2
8	American Journal of Chinese Medicine	12	1.54%	5.5	Q1
9	Drug Design Development and Therapy	12	1.54%	5.1	Q1
10	Journal of Pain Research	12	1.54%	2.5	Q2

**Figure 7:**
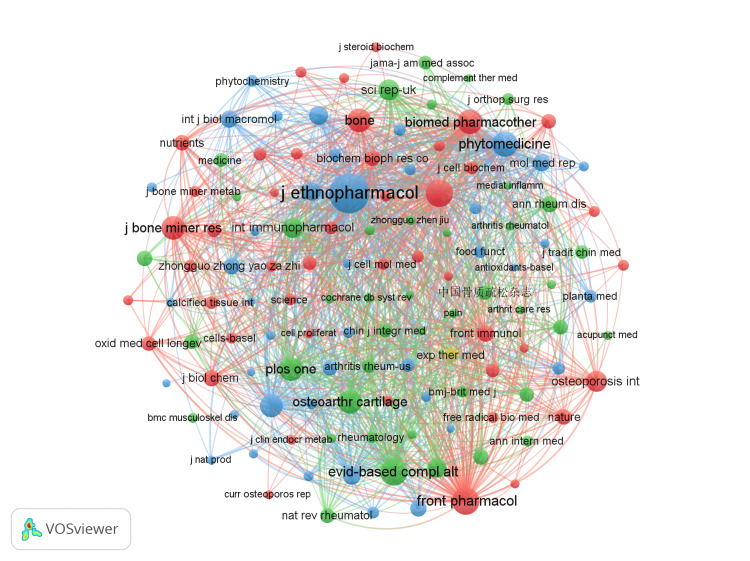
Citation network of magazines Citation Network was created by authors using VOSviewer (version 1.6.18).

The journal co-citation analysis is presented in Table 4 and Figure 7. The Journal of Ethnopharmacology was the most frequently co-cited journal (1341 citations), followed by Frontiers in Pharmacology (631 citations) and Evidence-Based Complementary and Alternative Medicine (628 citations). Among the top 10 co-cited journals, Osteoarthritis Cartilage had the highest impact factor (9.0), and 90% of the journals were classified as Q1 or Q2 journals.

**Table 4:** Cited references of journals IF - impact factor

Rank	Cited Journal	Co-citation	IF (2024）	Quartile in category
1	Journal of Ethnopharmacology	1341	5.4	Q1
2	Frontiers in Pharmacology	631	4.8	Q1
3	Evidence-Based Complementary and Alternative Medicine	628	-	Q3
4	International Journal of Molecular Sciences	627	4.9	Q2
5	Phytomedicine	548	8.3	Q1
6	Biomedicine & Pharmacotherapy	508	7.5	Q1
7	Osteoarthritis and Cartilage	495	9.0	Q1
8	PLoS One	487	2.6	Q2
9	Bone	474	3.6	Q2
10	Journal of Bone and Mineral Research	473	5.9	Q1

Authors and Co-cited Authors

**Figure 8:**
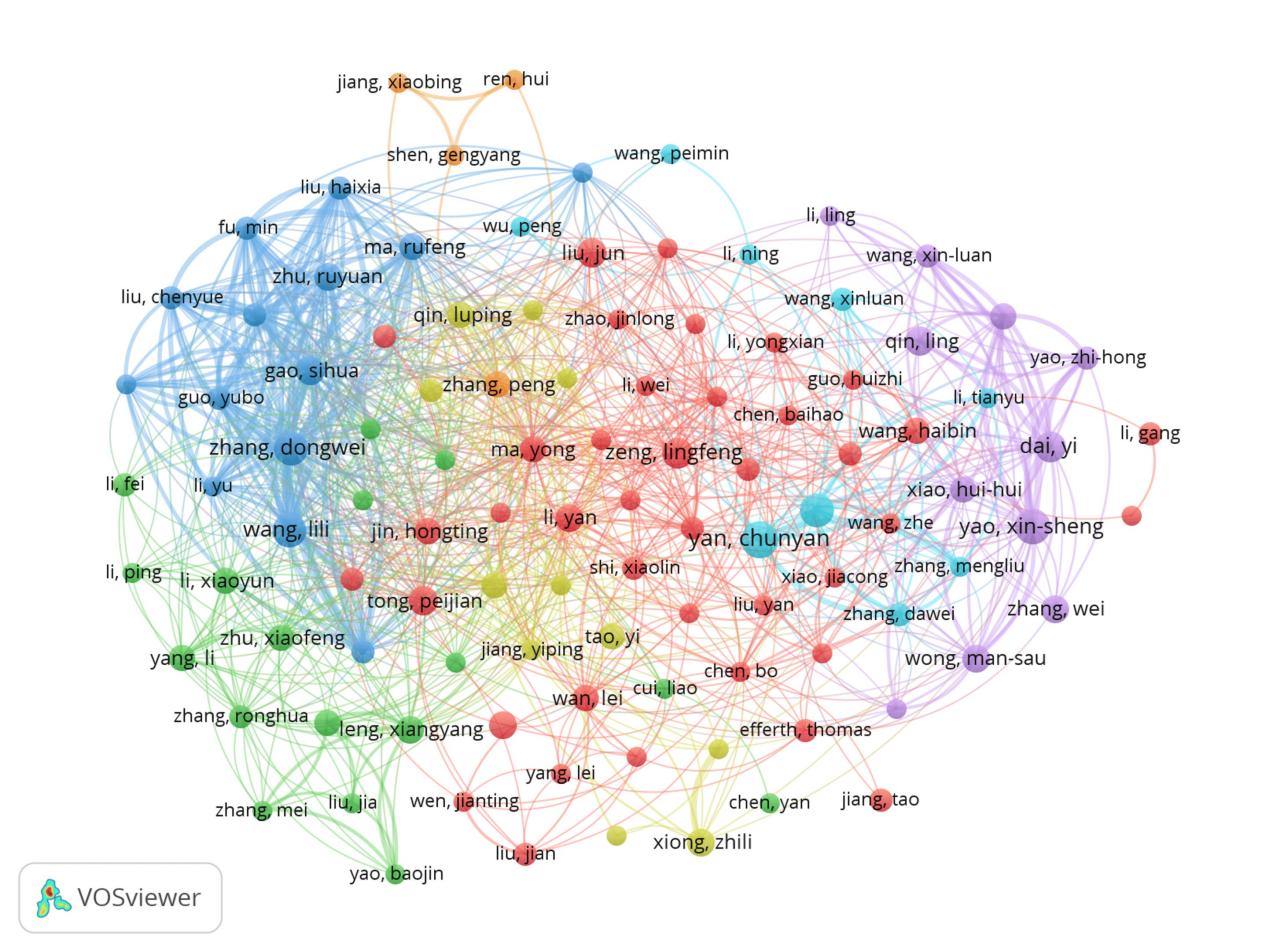
Author collaboration network diagram Diagram was created by the authors using VOSviewer (version 1.6.18).

Table 5 summarizes the 10 most productive authors and the 10 most co-cited authors in the field of TCM therapy for major musculoskeletal disorders. The 10 most productive authors contributed a total of 93 publications, accounting for 11.91% of all included studies. Chunyan Yan ranked first with 12 publications, followed by Xin-sheng Yao (11 publications), Lili Wang (10 publications), and Dongwei Zhang (10 publications). Figure 8 illustrates the collaboration network among authors, showing several relatively independent collaboration groups.

**Table 5:** Author publications and co-citations

Rank	Author	Count	Rank	Co-cited author	Citation
1	Chunyan Yan	12	1	Zhang Y	139
2	Xin-sheng Yao	11	2	Liu Y	105
3	Lili Wang	10	3	Wang Y	103
4	Dongwei Zhang	10	4	Li Y	99
5	Qian Zhang	9	5	Li J	90
6	Yi Dai	9	6	Zhang L	85
7	Lingfeng Zeng	8	7	Li X	74
8	Sihua Gao	8	8	Chen Y	68
9	Ling Qin	8	9	Liu J	66
10	Peijian Tong	8	10	Wang L	65

**Figure 9:**
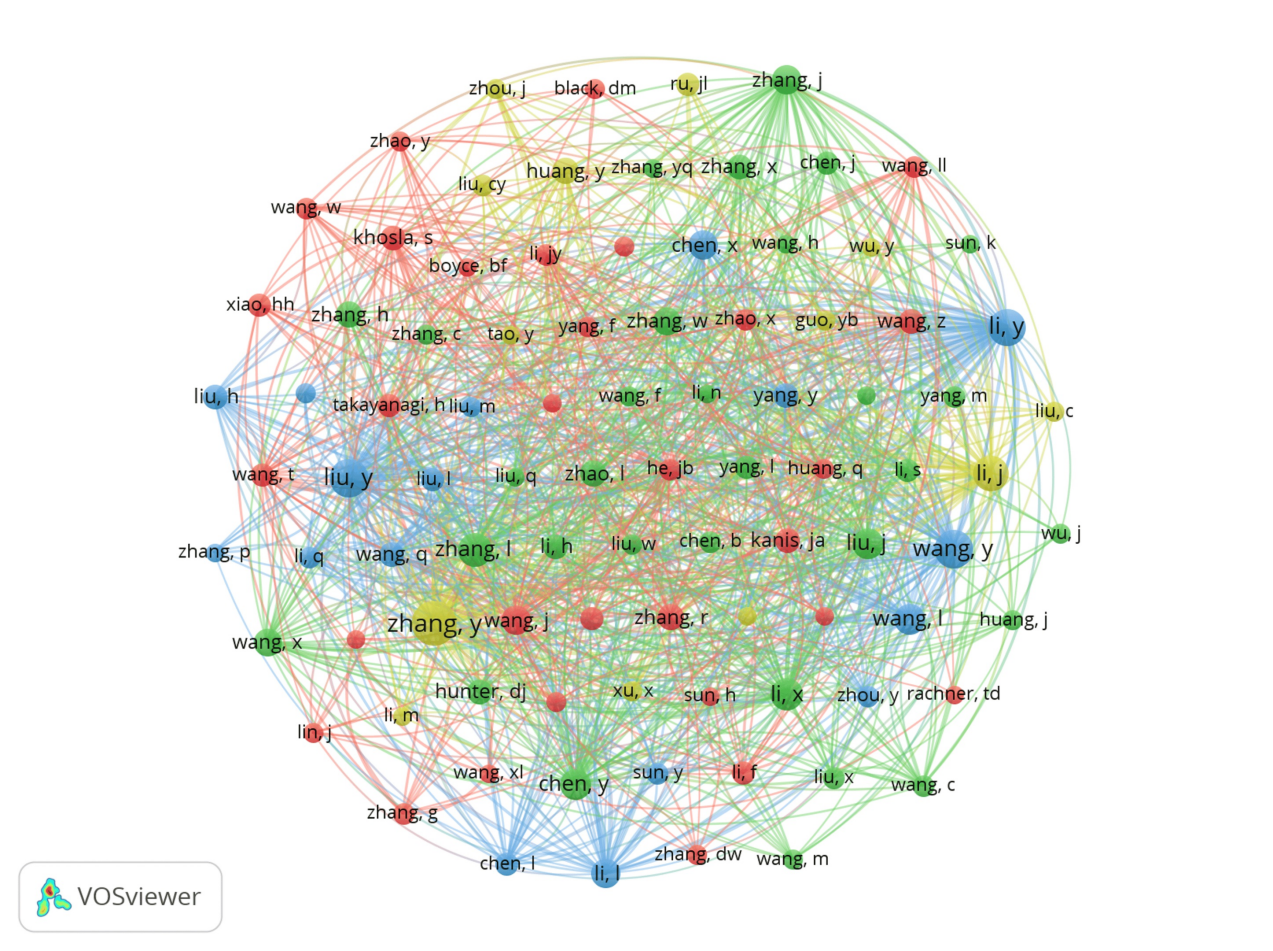
Author co-citation network diagram Diagram was created by the authors using VOSviewer (version 1.6.18).

The top three most co-cited authors were Zhang Y (139 citations), Liu Y (105 citations), and Wang Y (103 citations), indicating high citation impact within this research field. The author co-citation network is presented in Figure 9.

Co-cited References

**Figure 10:**
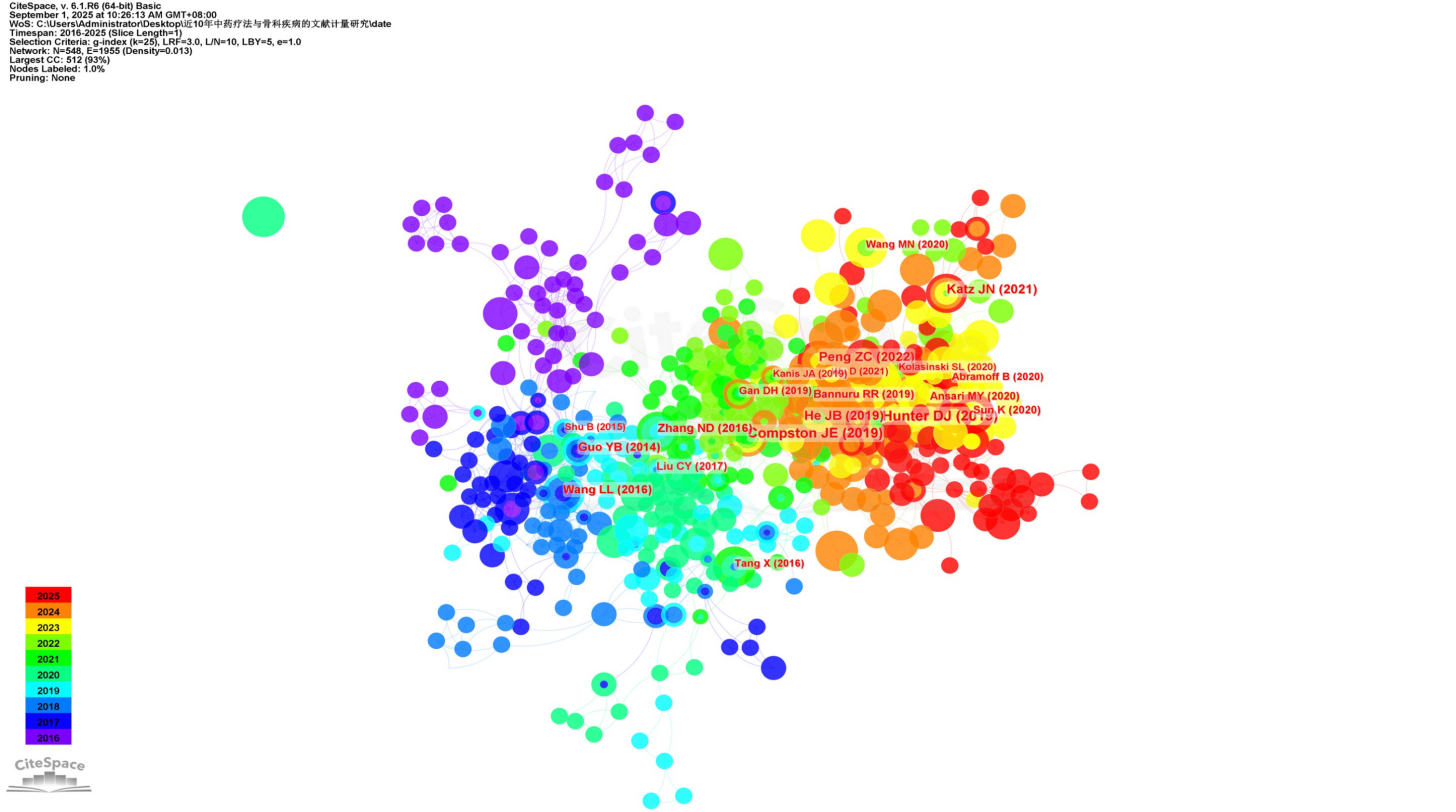
Network diagram of co-cited literature Diagram was created by authors using CiteSpace v.6.1.R6 (64-bit) Basic.

Co-citation analysis of references was performed using VOSviewer. The 10 most frequently co-cited references are listed in Table 6, and the corresponding co-citation network is shown in Figure 10. The three most frequently co-cited references were TCMSP: A Database of Systems Pharmacology for Drug Discovery from Herbal Medicines (40 citations), Traditional Chinese Medicine Formulas for the Treatment of Osteoporosis: Implications for Anti-osteoporotic Drug Discovery (39 citations), and Osteoarthritis (36 citations), indicating that database development, osteoporosis research, and osteoarthritis were the major knowledge bases in this field.

**Table 6:** Cited references in the literature

Rank	Title	Journal	Total citations	Reference
1	TCMSP: a database of systems pharmacology for drug discovery from herbal medicines	J Cheminform	40	Ru et al. [4]
2	Traditional Chinese medicine formulas for the treatment of osteoporosis: Implication for antiosteoporotic drug discovery	J Ethnopharmacol	39	Zhang et al. [5]
3	Osteoarthritis	Lancet	36	Hunter et al. [6]
4	Osteoclast differentiation and activation	Nature	28	Boyle et al. [7]
5	The first multicenter and randomized clinical trial of herbal Fufang for treatment of postmenopausal osteoporosis	Osteoporos Int	26	Zhu et al. [8]
6	Osteoporosis	Lancet	25	Ye et al. [9]
7	Osteoporosis: now and the future	Lancet	24	Rachner et al. [10]
8	Osteoarthritis: an update with relevance for clinical practice	Lancet	24	Bijlsma et al. [11]
9	Osteoarthritis	Lancet	23	Kloppenburg et al. [12]
10	Salvia miltiorrhiza: an ancient Chinese herbal medicine as a source for anti-osteoporotic drugs	J Ethnopharmacol	21	Guo et al. [13]

**Figure 11:**
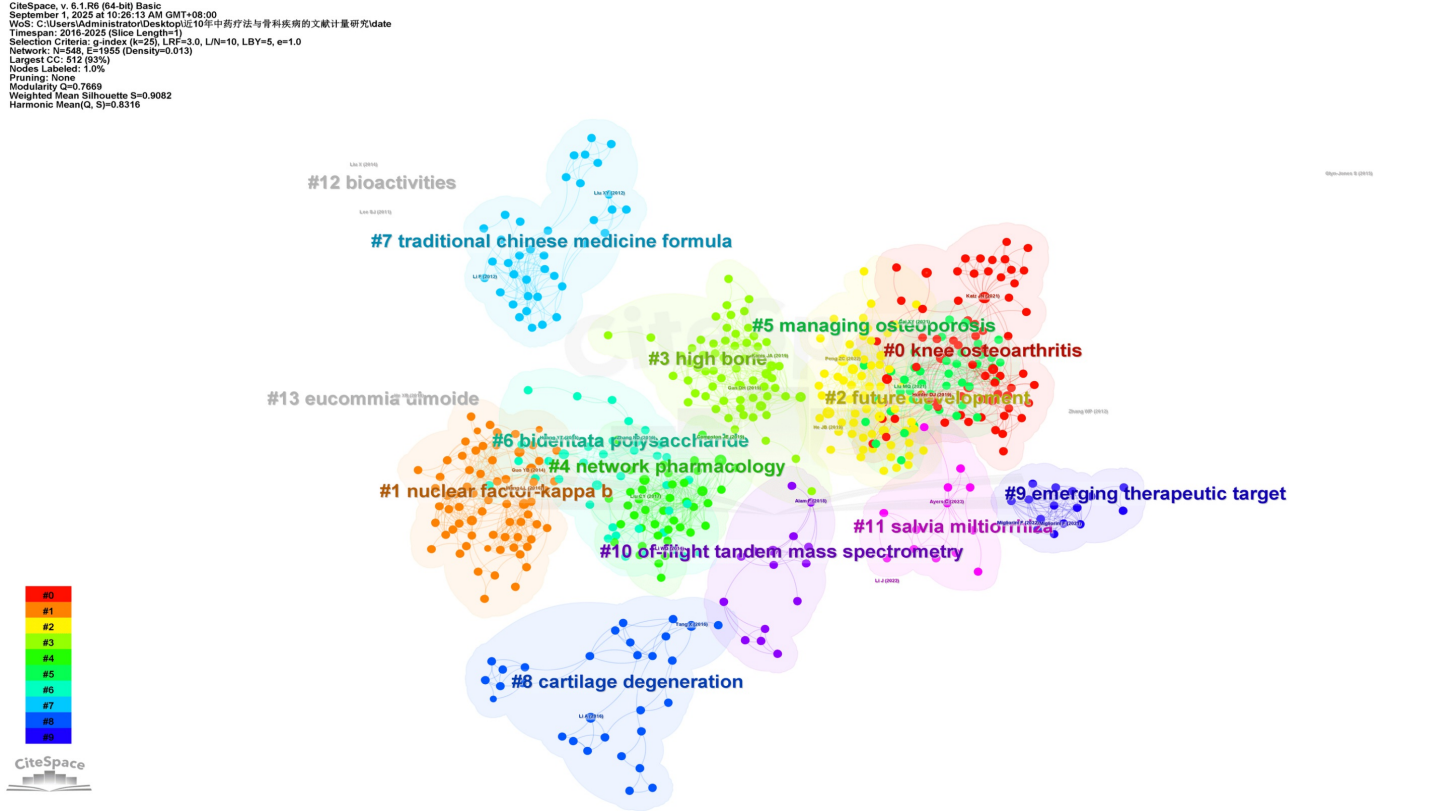
Cluster diagram of co-cited literature Diagram was created by authors using CiteSpace v.6.1.R6 (64-bit) Basic.

**Figure 12:**
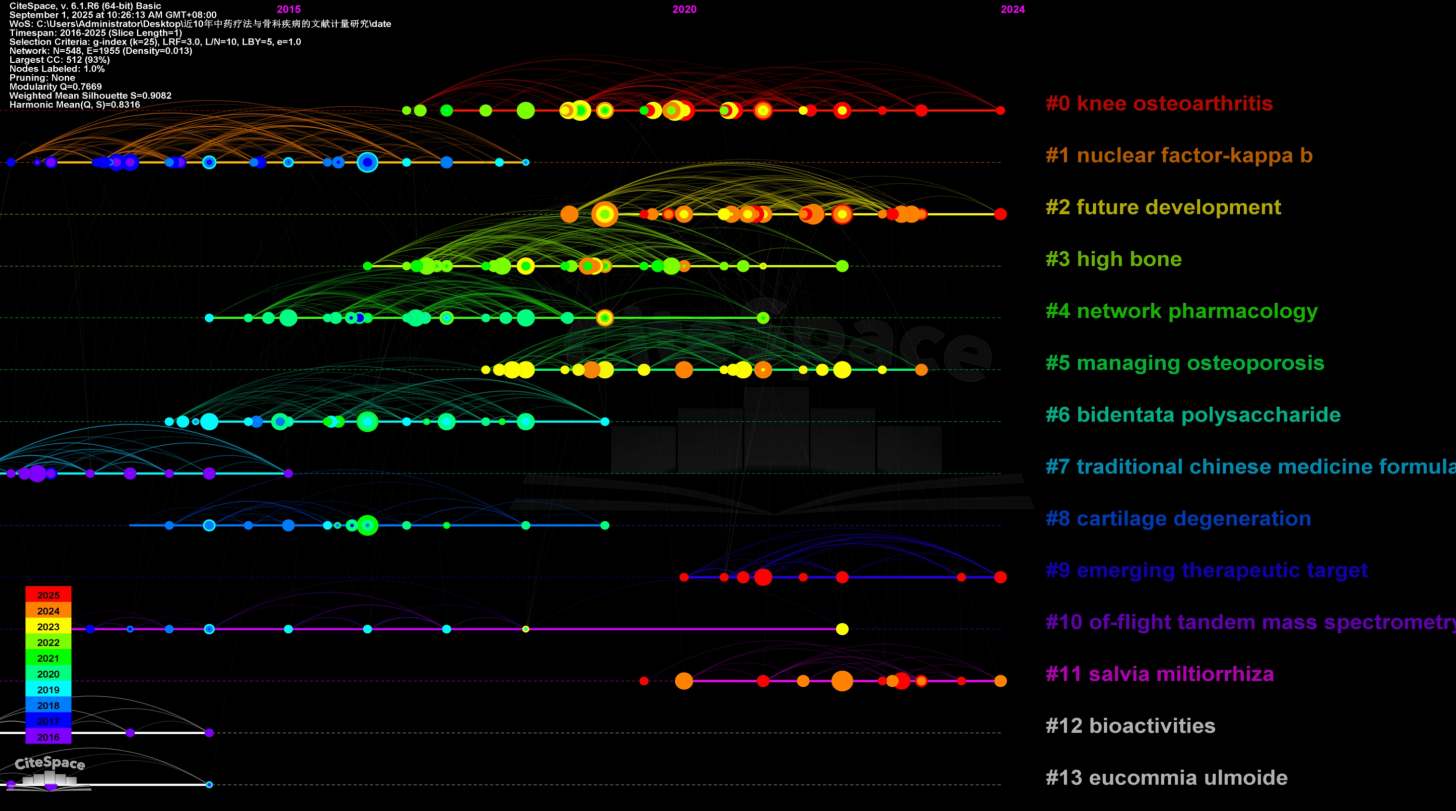
Timeline chart Chart was created by authors using CiteSpace v.6.1.R6 (64-bit) Basic.

Reference co-citation clustering and timeline analyses were further performed using CiteSpace (Figures 11 and 12). The clustering analysis generated a modularity Q value of 0.7669 and a weighted mean silhouette value of 0.9082, indicating a significant and reliable clustering structure. As shown in Figure 11, the major knowledge clusters included knee osteoarthritis (#0), nuclear factor-kappa B (NF-κB) (#1), network pharmacology (#4), managing osteoporosis (#5), and traditional Chinese medicine formula (#7), suggesting these were the major research topics in this field.

The timeline visualization (Figure 12) demonstrated the evolution of research hotspots from January 2016 to August 2025. Early studies mainly focused on single herbs (#6 and #13) and the NF-κB (#1). Since 2020, knee osteoarthritis (#0) has become the largest and most active research cluster. In addition, network pharmacology (#4) emerged around 2019 and became increasingly active after 2020. Since 2022, the emerging therapeutic target (#9) cluster has gradually emerged, reflecting recent research interests in TCM therapy for major musculoskeletal disorders.

Keyword Analysis

**Figure 13:**
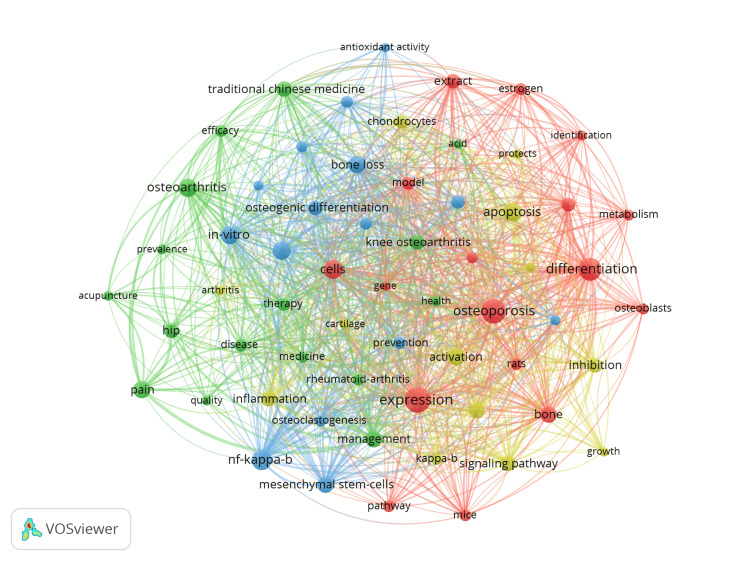
Network diagram of high-frequency keywords Diagram was created by authors using VOSviewer (version 1.6.18).

**Figure 14:**
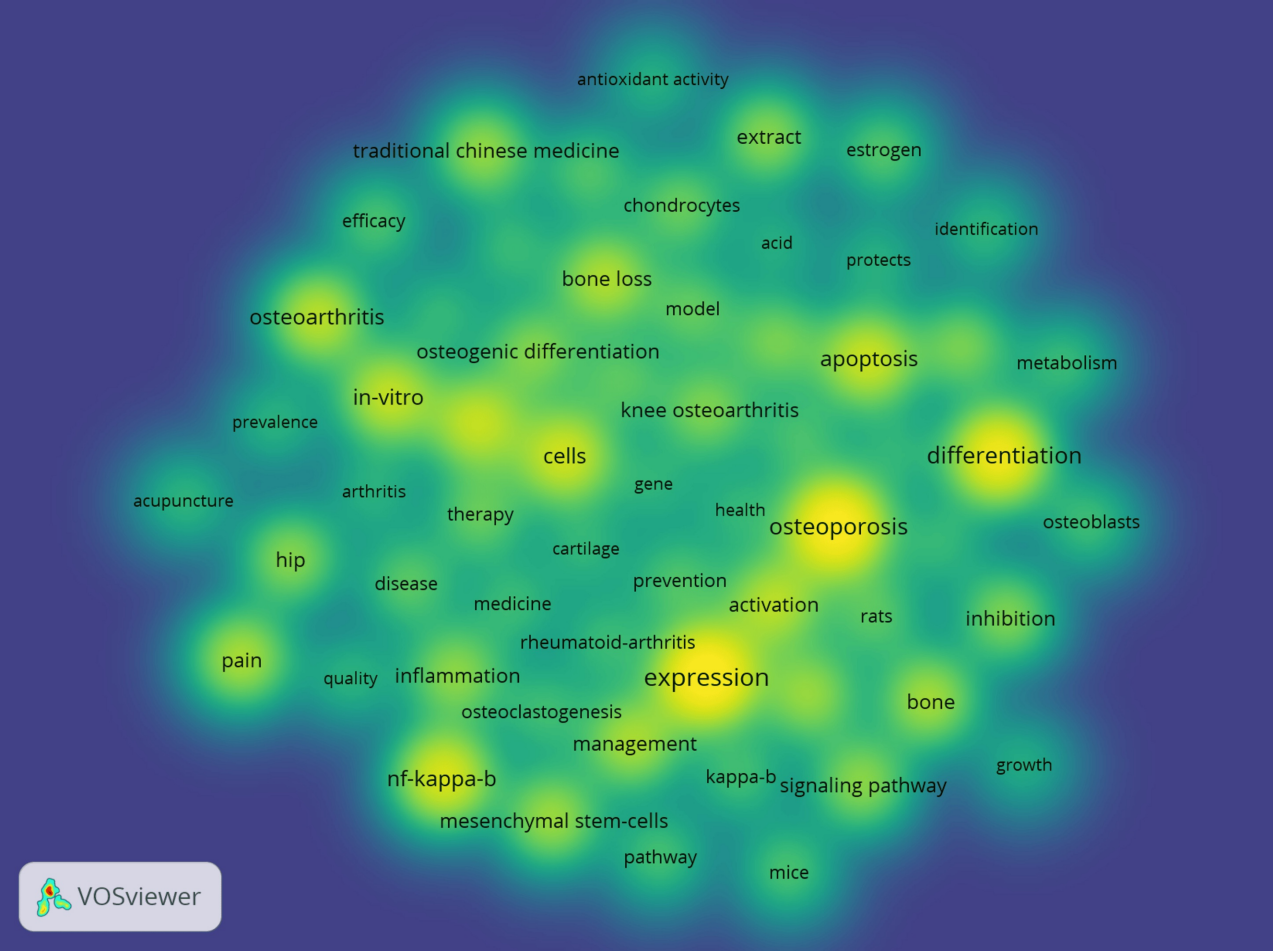
Keyword density chart Chart was created by authors using VOSviewer (version 1.6.18).

**Figure 15:**
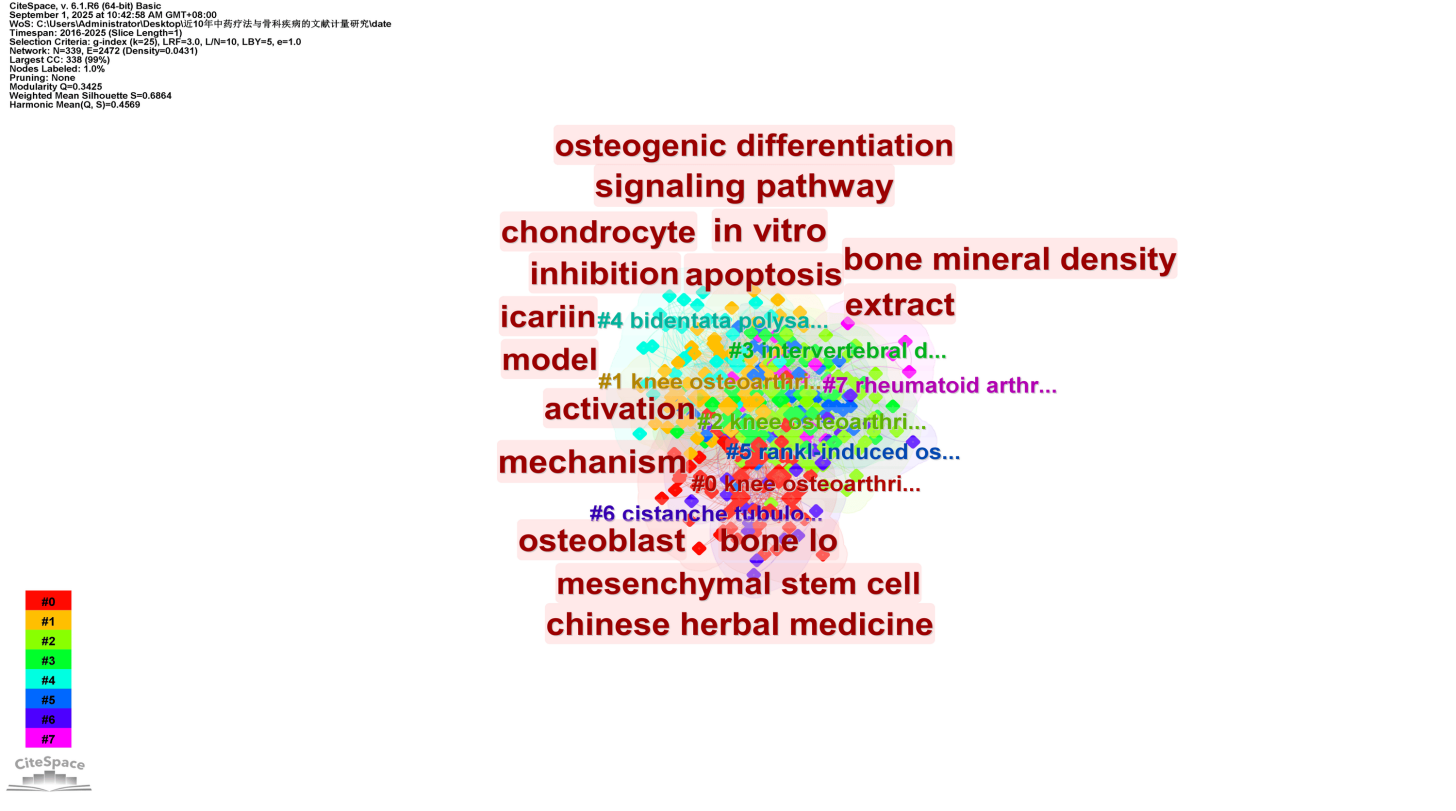
Keyword clustering diagram Diagram was created by authors using CiteSpace v.6.1.R6 (64-bit) Basic.

Keyword analysis was performed to identify the research hotspots and development trends in TCM therapy for major musculoskeletal disorders. The keyword co-occurrence analysis generated by VOSviewer identified "expression" (98 occurrences), "osteoporosis" (91), "differentiation" (76), and "NF-kappa-B" (61) as the most frequently occurring keywords (Table 7, Figures 13-14). After applying the occurrence threshold, a network consisting of 61 keywords was generated, which formed four major clusters. The keyword clustering analysis generated by CiteSpace is shown in Figure 15. The clustering network yielded a modularity Q value of 0.3425 and a mean silhouette value of 0.6864, indicating an acceptable clustering structure and consistency. The largest clusters were knee osteoarthritis (#0, #1, and #2), followed by RANKL-induced osteoclastogenesis (#5), Bidentata polysaccharide (#4), and Cistanche tubulosa (#6), demonstrating that osteoarthritis, osteoporosis, signaling pathways, and active components of TCM were the major research topics in this field.

**Table 7:** High-frequency keywords

Rank	Keyword	Counts
1	Expression	98
2	Osteoporosis	91
3	Differentiation	76
4	NF-kappa-b	61
5	Cells	55
6	Apoptosis	53
7	Oxidative stress	50
8	In-vitro	50
9	Osteoarthritis	48
10	Activation	46
11	bone loss	44
12	Pain	43
13	Management	42
14	Bone	41
15	Mesenchymal stem-cells	39
16	Proliferationt	37
17	Traditional Chinese medicine	37
18	Signaling pathway	35
19	Inflammation	35
20	Extract	34

Co-cited References and Keywords

**Figure 16:**
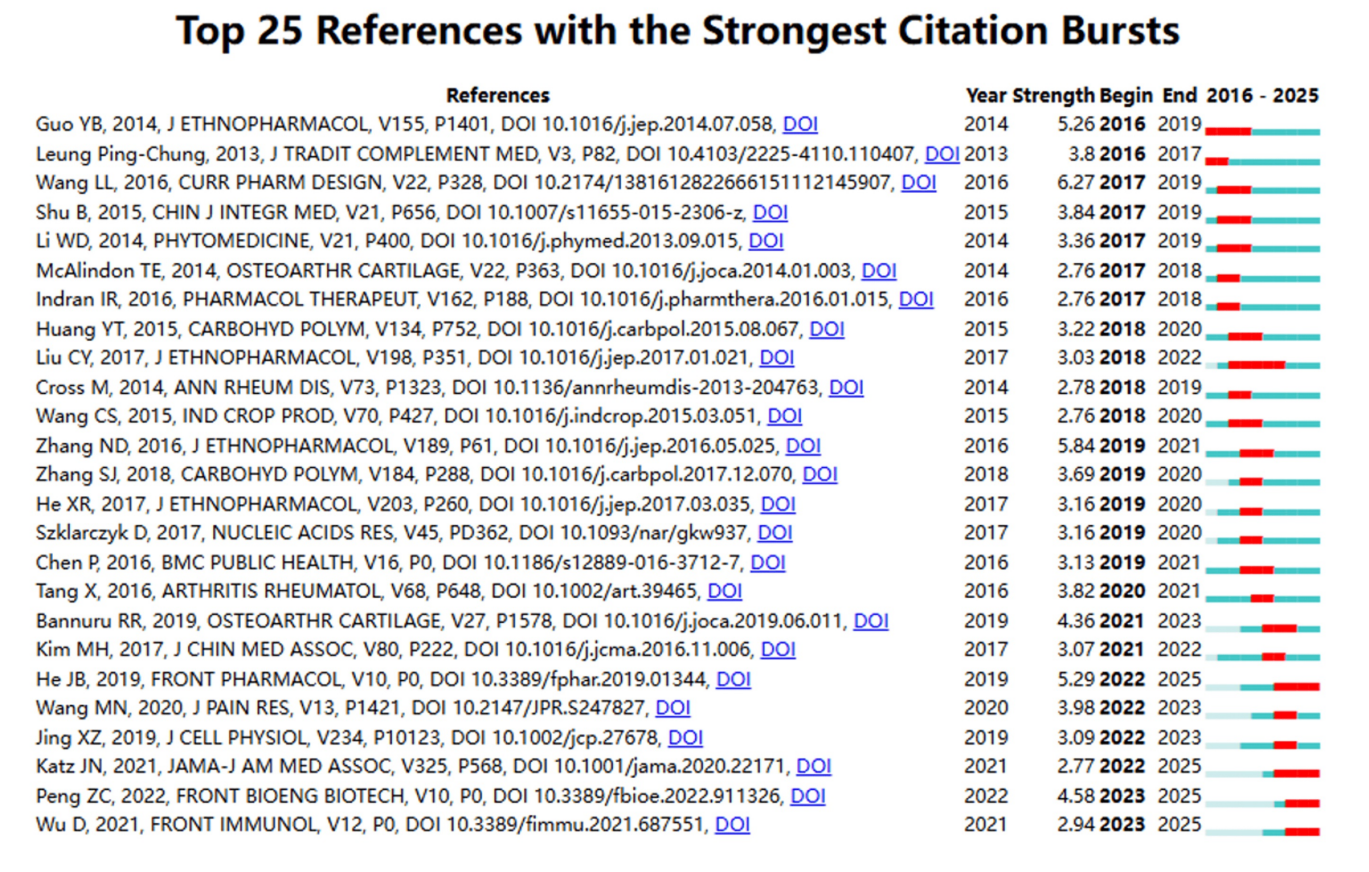
Citation literature emergence chart Chart was created by authors using CiteSpace v.6.1.R6 (64-bit) Basic. References: [5,13-36]

**Figure 17:**
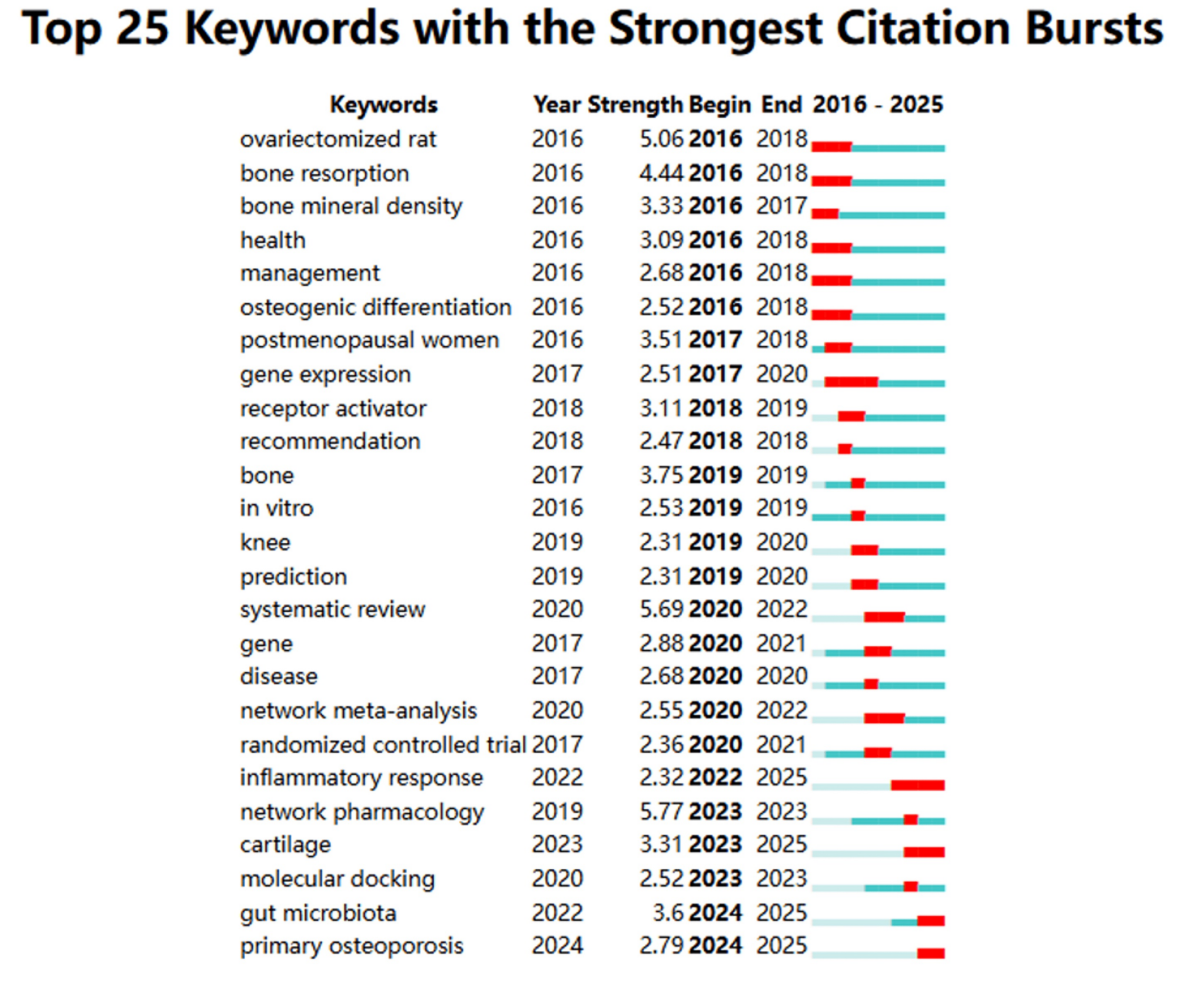
Keyword emergence diagram Diagram was created by authors using CiteSpace v. 6.1.R6 (64-bit) Basic.

The top 25 references and 25 keywords with the strongest citation bursts are presented in Figure 16 [5,13-36] and Figure 17, respectively. Citation burst analysis was performed to identify emerging research topics and changes in research interests over time.

Among the co-cited references, the two strongest citation bursts had burst strengths of 6.27 and 5.84, respectively. The strongest citation burst occurred between 2017 and 2019, whereas the second strongest burst occurred between 2019 and 2021, indicating changes in research emphasis during different periods.

The keyword citation burst analysis identified "network pharmacology" as the keyword with the highest burst strength (5.77), followed by several keywords related to osteoporosis research. Earlier burst keywords, including "ovariectomized rat" (5.06), "bone resorption" (4.44), "postmenopausal women" (3.51), and "bone mineral density" (3.33), were mainly associated with osteoporosis research. More recently, "network pharmacology" and "molecular docking" have emerged as the major burst keywords, reflecting the latest research trends in TCM therapy for major musculoskeletal disorders.

Discussion

This study provides a comprehensive bibliometric overview of global research on traditional Chinese medicine (TCM) therapy for major musculoskeletal disorders from January 2016 to August 24, 2025. By systematically analyzing publication outputs, contributing countries and institutions, influential journals and authors, co-cited references, and keyword networks, we characterized the knowledge structure, major research hotspots, and evolving trends in this field. Our findings demonstrated a continuous increase in research activity over the past decade, with China contributing the largest number of publications and the United States exhibiting greater international citation impact. In addition, osteoporosis, knee osteoarthritis, active compounds, signaling pathways, and network pharmacology were identified as the major research focuses, while molecular docking emerged as a recent research trend. These findings provide a comprehensive understanding of the current research landscape and may offer valuable guidance for future research and international collaboration.

The continuous increase in publications over the past decade reflects the growing research interest in TCM therapy for major musculoskeletal disorders. This trend may be attributed to the unique therapeutic characteristics of TCM, including its multi-component, multi-target, and holistic regulatory properties, which have demonstrated considerable potential in the prevention and treatment of major musculoskeletal diseases. In recent years, numerous studies have investigated the pharmacological activities of individual herbal compounds. For example, Icariin (ICA) promotes osteogenic differentiation by inducing Runx2 expression and activating the BMP signaling pathway [37], whereas Tetrandrine alleviates bone destruction in collagen-induced arthritis by inhibiting osteoclast genesis [38,39]. Meanwhile, increasing attention has been directed toward TCM formulas because of their synergistic therapeutic effects and relatively favorable safety profiles. Erxian Decoction has been reported to exert anti-osteoporotic effects by regulating carbonic anhydrase 2 and integrin β1 expression [40], while the Bushenhuoxue formula promotes osteogenic differentiation and inhibits osteoclast formation in osteoporosis and osteoarthritis [41,42]. These findings are consistent with our bibliometric results, which showed a gradual shift in research focus from single herbal medicines to TCM formulas over the past decade.

Our bibliometric analysis also revealed dynamic changes in national and institutional contributions to research on TCM therapy for major musculoskeletal disorders. China contributed the largest number of publications, whereas the United States demonstrated greater international citation impact, indicating its strong academic influence in this field. At the institutional level, collaborations were primarily established among institutions within the same country, while international cooperation remained relatively limited. This pattern suggests that although research productivity has increased substantially, global academic collaboration has not developed proportionally. Strengthening international cooperation may help overcome academic barriers, facilitate knowledge exchange, and promote the global development of TCM research for major musculoskeletal disorders. Future collaborations between high-output countries and leading research institutions may further enhance research quality, accelerate scientific innovation, and improve the international recognition of TCM.

The co-citation, keyword, and citation burst analyses together revealed the evolution of research hotspots and the knowledge structure of TCM therapy for major musculoskeletal disorders. Among the most frequently co-cited references, TCMSP - a database of systems pharmacology for drug discovery from herbal medicines and traditional Chinese medicine formulas for the treatment of osteoporosis and implications for anti-osteoporotic drug discovery - has provided important theoretical and methodological foundations for subsequent studies. The former established a systems pharmacology platform integrating herbal ingredients, targets, diseases, and pharmacokinetic properties, whereas the latter comprehensively summarized the pharmacological effects and mechanisms of TCM formulas for osteoporosis. These highly cited studies have substantially promoted mechanism-oriented research in this field.

Keyword cluster analysis further demonstrated that current research mainly focuses on osteoporosis, hip and knee osteoarthritis, active compounds, and signaling pathways. Mechanistic studies have particularly emphasized the regulation of bone metabolism through pathways associated with NF-κB, osteoblast differentiation, and osteoclast formation. These findings indicate that TCM research has gradually shifted from evaluating therapeutic efficacy toward elucidating the molecular mechanisms underlying major disorders.

Citation burst analysis further illustrated the changing research priorities over time. Before 2020, studies mainly focused on the therapeutic effects, active compounds, and mechanisms of single Chinese traditional medicines for osteoporosis, including *Epimedii*, *Salvia miltiorrhiza*, *Psoraleae corylifoliae*, *Rehmanniae Radix*, and *Achyranthes bidentata*. However, after 2020, increasing attention has been directed toward TCM formulas, while hip and knee osteoarthritis have gradually become major research hotspots. More recently, network pharmacology and molecular docking have emerged as the strongest burst keywords, highlighting the growing application of computational approaches for investigating the pharmacological mechanisms of TCM. These findings suggest that current research is evolving from empirical evaluation of herbal medicines toward mechanism-based and systems-level investigations.

Based on the identified research hotspots and emerging trends, several future research directions deserve further attention. First, network pharmacology and molecular docking have become increasingly important computational tools for elucidating the complex mechanisms underlying TCM therapy for major musculoskeletal disorders. The integration of these approaches with artificial intelligence (AI) and multi-omics technologies is expected to improve target prediction, drug screening, and mechanism discovery, thereby accelerating the modernization of TCM research. Second, although the study has mainly focused on osteoporosis, hip osteoarthritis, and knee osteoarthritis, research on other musculoskeletal disorders, including spinal diseases (such as cervical spondylosis, lumbar disc herniation, scoliosis, and fractures) and muscle diseases (such as muscle atrophy and myositis), remains limited and warrants further investigation. Finally, strengthening interdisciplinary collaboration among clinicians, pharmacologists, bioinformaticians, and data scientists, together with expanding international cooperation, may facilitate the translation of basic research into clinical applications and promote the global development of TCM for major musculoskeletal disorders.

Limitations

This study has several limitations that should be acknowledged. First, although the Web of Science Core Collection (WoSCC) was selected because of its high-quality indexing and standardized bibliographic records, relevant publications indexed in other databases were not included, which may have resulted in an incomplete representation of the global research landscape. Second, this bibliometric analysis focused on major musculoskeletal disorders and, therefore, may not fully capture research related to less common or emerging musculoskeletal conditions. Future studies incorporating a broader range of diseases and multiple literature databases may provide a more comprehensive overview of this field. Third, bibliometric analysis provides a quantitative assessment of research activities but cannot fully evaluate the scientific quality or clinical significance of individual studies. Finally, although the search strategy was carefully designed and the complete search syntax has been provided in the Appendix to improve transparency and reproducibility, some recently emerging terminology or disease-specific keywords may not have been fully represented.

## Conclusions

Based on publications retrieved from the Web of Science Core Collection between January 2016 and August 24, 2025, this study systematically characterized the global research landscape of traditional Chinese medicine (TCM) therapy for major musculoskeletal disorders using bibliometric methods. The results demonstrated a continuous increase in research activity over the past decade and identified China and the United States as the leading contributors to this field. Osteoporosis, knee osteoarthritis, active compounds, and molecular signaling pathways represented the major research hotspots, whereas network pharmacology and molecular docking have emerged as important methodological trends, reflecting a shift from evaluating therapeutic efficacy toward mechanism-oriented research.

Overall, this study provides a comprehensive overview of the knowledge structure, research hotspots, and evolving trends in TCM research for major musculoskeletal disorders. Our findings offer valuable references for researchers, promote international academic collaboration, and may facilitate the integration of traditional Chinese medicine with modern computational approaches and translational research, thereby supporting the future development of this field.

## Appendices

**Table 8:** Appendix 1 - Complete search strategy The search strategy was designed to capture major musculoskeletal disorders that are most frequently investigated in relation to traditional Chinese medicine and was consistently applied throughout the bibliometric analysis.

Item	Description
Database	Web of Science Core Collection (WoSCC)
Search date	August 24, 2025
Search period	January 1, 2016 – August 24, 2025
Search field	Topic (TS)
Language	English
Document types	Articles and Reviews
Search query	TS=((“orthopedic diseases” OR “osteoarthritis” OR “osteoporosis” OR “bone fracture” OR “spinal stenosis” OR “intervertebral disc degeneration”) AND (“traditional Chinese medicine” OR TCM OR “Chinese herbal medicine”))
Records retrieved	781
Duplicate records	0
Final records included	781
